# Characterization of the microheterogeneity of transthyretin in plasma and urine using SELDI-TOF-MS immunoassay

**DOI:** 10.1186/1477-5956-2-5

**Published:** 2004-09-01

**Authors:** Florian J Schweigert, Kerstin Wirth, Jens Raila

**Affiliations:** 1 Department of Physiology and Pathophysiology, Institute of Nutritional Science, University of Potsdam, A.- Scheunert-Allee 114-116; D-14558 Potsdam-Rehbrücke, Germany

## Abstract

**Background:**

It has been shown that transthyretin (TTR) exists in different molecular variants. Besides point mutations associated with different diseases such as amyloidosis, other posttranslational modifications occur that might be of diagnostic interest.

**Results:**

TTR levels as determined by ELISA in plasma and urine of healthy individuals were 489 ± 155 μg/ml plasma and 46 ± 24 ng/g creatinine, respectively. Average levels in urine of pregnant women were 45 ± 65 μg/g creatinine. The molecular heterogeneity of TTR was analyzed using a high-throughput mass spectrometric immunoassay system. TTR was extracted from plasma or urine onto an antibody-coated (via protein A) affinity chip surface (PS20) using the surface-enhanced laser desorption/ionization (SELDI) technique. Subsequently samples were subjected to time-of-flight mass spectrometry (TOF-MS). In healthy individuals, TTR in plasma occurred rather consistently in two variants of 13732 ± 12 and 13851 ± 9 Da for the native and S-cysteinylated forms and at a smaller signal of 14043 ± 17 Da for the S-glutathionylated form. In urine of pregnant women, various signals were observed with a dominant signal at 13736 ± 10 Da and a varying number of smaller immunoreactive fragments. These fragments are possibly the consequence of metabolism in plasma or kidney.

**Conclusion:**

This chip-based approach represents a rapid and accurate method to characterize the molecular variants of TTR including protein or peptide fragments which are either related to TTR or have resulted from its catabolism. These molecular variants may be of diagnostic importance as alternative or novel biomarkers due to their predominant relation to the TTR metabolism both in healthy and diseased individuals.

## Background

Transthyretin (TTR, formerly called prealbumin) belongs to a group of proteins including thyroxine-binding globulin and albumin which bind and transport thyroid hormones in the blood. It is a single polypeptide chain of 127 amino acids (14 kDa) and is present in the plasma as a tetramer of non-covalently bound monomers. The major sites of TTR synthesis are the liver and choroid plexus [1-3]. Under physiological conditions, the macromolecular complex plays an important physiological role in vitamin A homeostasis because it binds the specific transport protein for retinol, the lipocalin retinol-binding protein (RBP) [4,5]. This reduces the glomerular filtration of the low molecular weight transport protein (21 kDa) in the kidneys. Any TTR or RBP molecules that are filtered are rapidly bound to megalin, the multiligand receptor expressed on the luminal surface of the renal proximal tubules and therefore internalized. Thus, under physiological conditions, TTR and RBP are present in urine if at all, only in trace amounts [6].

The TTR variants described thus far have mostly been associated with variable degrees of cardiac and/or neural tissue amyloid deposits [7,8]. Therefore, mutations of the amino acid sequence of TTR are of clinical interest [9]. In general, mutations appear to be distributed randomly within the molecule and most of these mutations lead to the synthesis of TTR molecules which have the tendency to form insoluble protein aggregates. These so-called amyloid deposits accumulate extracellularly in various organs. Although the role of amyloid deposits in the pathogenesis of the disease is not clear, preventing their formation or promoting their disaggregation is necessary to control the development of clinical symptoms [10,11].

With regard to nutrition, TTR is a so-called visceral protein that is synthesized in the liver in response to nutritional supply. TTR plasma levels have thus been proposed as sensitive biochemical parameters of subclinical protein malnutrition, because both the adequacy and levels of protein as well as energy intakes are reflected in plasma levels. Plasma levels of TTR, however, are as well affected by acute and chronic diseases associated with an acute-phase response. Under these conditions, liver activity is converted to the synthesis of acute-phase response proteins, resulting in a dramatic drop in visceral proteins, despite nutritional support [1-3].

This study was conducted to establish a sensitive and reproducible high-throughput SELDI-TOF-MS immunoassay for characterizing TTR variants in plasma and urine arising from amino acid substitutions, posttranslational modifications and/or products of protein degradation or proteolysis.

## Results

### TTR levels in plasma and urine

TTR levels in plasma determined by ELISA in 10 healthy individuals were 489 ± 155 μg/ml. TTR levels in urine of the healthy individuals were one thousand times lower than in the 40 pregnant women with 46 ± 24 ng/g creatinine and 45 ± 65 μg/g creatinine, respectively.

### TTR microheterogeneity in plasma and urine

**Figure 1:**
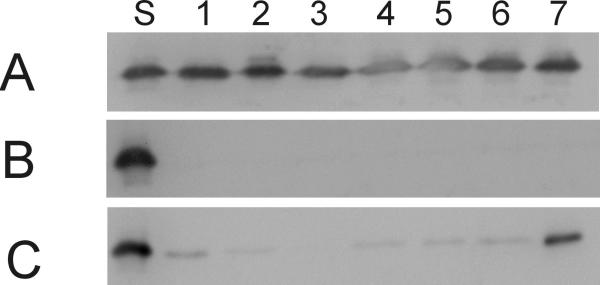
Western blot of TTR in paired plasma (A) and urine (B) of seven (1–7) healthy individuals and in seven urine samples (C) of healthy pregnant women. S = TTR standard

In Figure 1 the TTR variants found in serum and in urine of pregnant women and non-pregnant controls were compared using the Western blot technique. The results obtained with this method showed that TTR was present in plasma of both groups and in urine of pregnant women but not in the urine of non-pregnant individuals. The intensity of the immunoreactive band in urine of pregnant women was substantially lower than that in plasma. In all investigated TTR-positive plasma and urine samples TTR was present as a single band indicating a molecular weight of 14 kDa.

**Figure 2:**
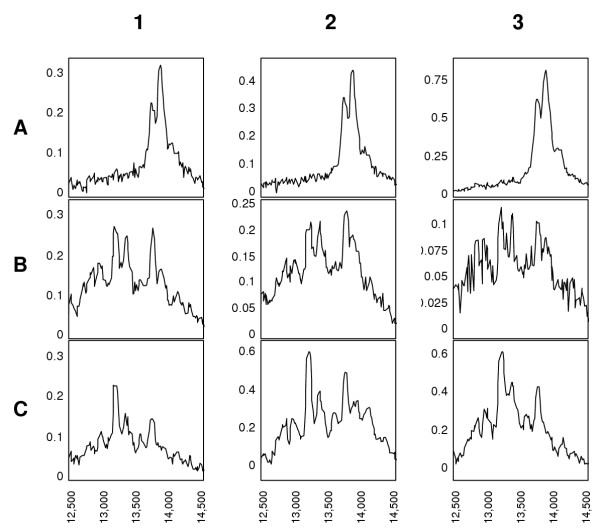
Mass spectra resulting from SELDI-TOF-MS immunoaffinity analysis of three (1–3) paired plasma (A) and urine (B) samples obtained from healthy individuals (a) and three urine samples (C) from healthy pregnant women.

SELDI-TOF-MS immunoassays showed that in healthy non-pregnant and pregnant females as well as in males TTR in plasma occurred rather consistently in two major variants of 13732 ± 12 and 13851 ± 9 Da, of which generally the 13851 Da variant is the dominant one. The mass difference between these two variant was 120 ± 9 Da. In addition to the two major signals a minor one at 14043 ± 17 Da was observed in plasma. The mass difference to the signal at 13851 Da was 192 ± 11 Da. In urine, traces of TTR were detected in samples from non-pregnant females and from males. In the urine of pregnant women however strong mass signals were observed. As shown in Figure 2c, the variant of 13736 ± 10 Da also present in plasma, as well as lower molecular immunoreactive species were observed at 12847 ± 29, 12984 ± 7, 13202 ± 16, 13349 ± 15 and 13575 ± 15 Da. In urine, although the TTR pattern was different to the one in plasma, there were similarities in all samples in which TTR was present.

## Discussion

The SELDI platform can be readily adapted for developing an immunoassay format. This approach, using antibodies as an affinity capture device has been successfully used to detect and quantify different proteins by MS in complex biological mixtures [12]. Unlike current ELISA technology that uses an indirect detection mechanism by "sandwiching" the targeted protein with antibodies, the SELDI-based immunoaffinity assay allows for direct detection without tags. The specificity of the mass spectrometry immunoassay is unsurpassed because there is the combined discriminating power of both the antibody and the high molecular weight accuracy of the detector. In terms of sensitivity, the detection limit can approach that of current assays for TTR. The antibody used as bait recognizes an epitope in different isoforms of TTR. This direct sampling is a unique advantage over the more traditional ELISA methods in which the resulting signal is a weighted average of all the bound species. The discriminating power of the assay is shown in the direct comparison between Western blotting (Figure 1) and SELDI-TOF-MS immunoassay (Figure 2). The small mass differences observed with mass spectrometry can not be resolved by the combination of electrophoresis and immunological detection.

The results support and confirm previous studies with regard to molecular variants of TTR in plasma and urine [13-15]. As in these studies, TTR in plasma was dominant in two variants. The 120 Da larger variant is the S-cysteinylated form of TTR. The inconsistently present smaller signal at 14043 Da can be attributed to the S-glutathionylated form of TTR [13,16,17]. Other forms due to dehydration, or phosphorylation were not observed. The reported isoforms of TTR result when the Cys10 residue makes a mixed disulfide with the amino acid cysteine, the peptide glutathione or the peptide cysteinyl-glycine. Recent studies show that the most prevalent modification of TTR renders it substantially more amyloidogenic than the non-cysteinylated form at pH 5 [18]. The possible importance as a risk factor for the onset of senile systemic amyloidosis remains to be elucidated. Additionally, the S-homocysteinylation of TTR has been described in plasma of humans with hyperhomocysteinemia [19]. These two aspects might support the importance of a diagnostic approach to characterize isoforms of TTR more easily.

Apart from the case of absence, some modifications might not be detected with this method due to limitations in resolution. Both methods, ELISA and SELDI immunoassay are sensitive enough to observe only trace amounts of TTR in body fluids as in the case of urine of healthy individuals. These amounts were not detected using immunoblotting. Despite the small signal it was obvious that additional variants to the ones in plasma are present in urine. Contrary to a pervious study using a different method [14] we were not able to observe the cysteinylated form of TTR in urine despite being the dominant one in paired plasma samples. Major differences to this method are the possibility of one step on chip-enrichment and the analysis of much smaller sample amounts but a lower mass resolution. This latter aspect however, can not explain these differences.

No study is available in which TTR has been described quantitatively in the urine of healthy individuals. In this study we are able to show for the first time that levels of TTR in urine of pregnant women are 1000-fold higher compared to the levels in urine of healthy non-pregnant individuals. The excretion of substantial amounts of TTR has only been described in individuals with different kidney diseases indicating disturbed glomerular filtration and/or insufficient tubular reabsorption [13]. The involvement of the megalin receptor in the tubular reabsorption of filtered TTR has been shown in patients with Dent's disease [6].

Interestingly, as shown by us also for the first time, TTR in the urine of pregnant women is present in different molecular variants. In contrast to plasma, the S-cysteinylated form dominant in plasma was if at all present only in trace amounts. It remains to be determined if this is limited to the excretion of TTR in pregnancy or if under other circumstances such as in different kidney diseases similar molecular variants occur as well. The lower molecular weight immunoreactive variants found in urine of pregnant women may arise from limited proteolysis. At this point it is not possible to determine location and extent of proteolysis within the molecule. The immunoreactive fragments might arise from a breakdown within the plasma or the kidney structures.

## Conclusions

The SELDI immunoaffinity-isolation of TTR in combination with mass spectrometry offers a rapid, highly reproducible and cost-effective system for the determination of molecular variants of microheterogenous proteins and peptides. This is of importance in "second phase" proteomics which is characterized by the repetitive investigation of the same protein to validate the protein phenotype in large population based studies. This provides the basis for a substantial progress in the diagnostic with regard to personalized medicine [20,21]. With regard to TTR, this approach might not only be of importance in the diagnosis of TTR related amyloidosis but may also have the potential as alternative or new biomarkers related to the metabolism of TTR for use in nutrition related disease as well as in the diagnosis of kidney function.

## Methods

### Participants

Paired plasma and urine samples from 10 healthy individuals were obtained. The plasma was prepared by centrifugation of the blood (1500 × *g*, 10 min, 4°C) within 1–3 h after acquisition. Additionally, urine samples of the ten individuals and of healthy pregnant women (n = 40) undergoing routine medical examination, were collected and immediately mixed with protease inhibitors (Sigma, Deisenhofen, Germany). Hematuria was tested using a routine dipstick method (Combur 9 Test, Roche, Basel, Switzerland). Cells and other non-soluble material were cleared from the sample by brief centrifugation (1500 × *g*, 2 min). Aliquots of centrifuged urine were stored at -80°C and processed as soon as possible. The study protocol was approved by the hospitals and University of Potsdam Ethics Committee. Informed consent was obtained from each participant.

### Determination of TTR using ELISA and Western blotting

Polyclonal antibodies to TTR were produced in rabbit and affinity purified (Dako Diagnostics, Hamburg, Germany). TTR in plasma and urine was quantitatively determined by an enzyme-linked immunoassay (ELISA) method developed in our laboratory. To further assess the presence of TTR quantitatively and qualitatively, we performed a SDS-polyacrylamide gel electrophoresis (PAGE) immunoblot analysis as has been described [22].

### Immuno SELDI-TOF-MS

Protein A (Sigma, Deisenhofen, Germany) at 0.1 mg/ml in PBS was added (5 μl) to the spots of a pre-activated ProteinChip® Array (PS20; Ciphergen Biosystems Inc., Palo Alto CA, USA). The PS20 array consists of a surface with epoxy groups that dock proteins by covalently reacting with their amine groups. The arrays were incubated for 1 h at 25°C in a humidity chamber. After blocking residual active sites with 5 μl blocking buffer (0.5 M ethanolamine in PBS at pH 8.0) for 25 min, the array was washed three times in a 15 ml conical tube with PBS (pH 7.4). TTR antibody (4.1 μg/μl) was added to individual spots (2 μl) and incubated in a humidity chamber for 1.5 h with mixing. The unbound antibodies were removed by washing the array three times in a 15 ml conical tube with PBS (pH 7.4).

Before incubation urine was diluted 1:1 (v:v) in PBS and plasma 1:100 (v:v) in PBS. A total volume of 10 μl was applied to each spot and incubated for 1.5 h at 25°C in a humidity chamber. Samples were washed on the spot with 5 μl PBS three times and finally with 5 μl H_2_O. After drying, 0.6 μl of a saturated energy-absorbing molecule (EAM) solution (5 mg sinapinic acid dissolved in 75 μl acetonitrile and 75 μl 1% trifluoroacetic acid) was applied to the spot surface, and the sample was allowed to dry.

After insertion of the ProteinChip Array into the ProteinChip Reader, a laser pulse was focused on the sample under vacuum. The array was analyzed under the following settings: laser intensity 250/220, detector sensitivity 8, mass focus, molecular mass range 10 to 18 kDa, position 20–80 and a 150–200-shot average per sample.

CytochromeC (equine cardiac; 12360.1 MW), myoglobin (equine cardiac; 16951.5 MW), GAPDH (rabbit; 35688 MW), albumin (bovine serum; 66433MW) and β-galactosidase (E. coli; 116351MW) were used as calibrators.

Mass resolution (defined as m/Δm) is routinely achieved below 300 and mass accuracy was within 0.1%. Peaks with amplitudes at least 3 times greater than the average background noise level were considered. The reproducibility was tested with different aliquots of the same sample on eight different spots of the protein chip array.

## Competing interests

None declared.

## Authors' contributions

FJS participated in the conception, design, data analysis and the writing of the manuscript, KW in data analysis and writing of parts of the manuscript. JR participated in the conception, data analysis and the writing of the manuscript. All authors have read and approved the last version of the manuscript.
